# Interhemispheric Plasticity following Intermittent Theta Burst Stimulation in Chronic Poststroke Aphasia 

**DOI:** 10.1155/2016/4796906

**Published:** 2016-01-10

**Authors:** Joseph C. Griffis, Rodolphe Nenert, Jane B. Allendorfer, Jerzy P. Szaflarski

**Affiliations:** ^1^Department of Psychology, University of Alabama at Birmingham, Birmingham, AL 35294-0021, USA; ^2^Department of Neurology, University of Alabama at Birmingham, Birmingham, AL 35294-0021, USA; ^3^Department of Neurology, University of Cincinnati Academic Health Center, Cincinnati, OH, USA

## Abstract

The effects of noninvasive neurostimulation on brain structure and function in chronic poststroke aphasia are poorly understood. We investigated the effects of intermittent theta burst stimulation (iTBS) applied to residual language-responsive cortex in chronic patients using functional and anatomical MRI data acquired before and after iTBS. Lateralization index (LI) analyses, along with comparisons of inferior frontal gyrus (IFG) activation and connectivity during covert verb generation, were used to assess changes in cortical language function. Voxel-based morphometry (VBM) was used to assess effects on regional grey matter (GM). LI analyses revealed a leftward shift in IFG activity after treatment. While left IFG activation increased, right IFG activation decreased. Changes in right to left IFG connectivity during covert verb generation also decreased after iTBS. Behavioral correlations revealed a negative relationship between changes in right IFG activation and improvements in fluency. While anatomical analyses did not reveal statistically significant changes in grey matter volume, the fMRI results provide evidence for changes in right and left IFG function after iTBS. The negative relationship between post-iTBS changes in right IFG activity during covert verb generation and improvements in fluency suggests that iTBS applied to residual left-hemispheric language areas may reduce contralateral responses related to language production and facilitate recruitment of residual language areas after stroke.

## 1. Introduction

Strokes of the left middle cerebral artery (LMCA) territory often lead to impairments in language function that are collectively referred to as aphasias [1]. Language recovery after LMCA stroke is highly variable, and many patients remain chronically aphasic despite optimal rehabilitative approaches [2, 3]. Aphasia following LMCA stroke typically results from lesions affecting frontal and/or temporal language regions in the left hemisphere and also often involves damage to white matter pathways connecting these regions [4–11].

Functional neuroimaging studies indicate that the recovery of language abilities after LMCA stroke involves the restoration of language-related processing in the remaining tissues near affected language areas as well as the compensatory recruitment of unaffected areas for language-related processing [12–14]. While downregulated responses in affected left-hemisphere language areas and upregulated responses in unaffected right-hemisphere homologues are commonly observed during language task performance in acute patients [12, 13, 15], the restoration of typical language-related responses in residual left-hemisphere language areas (which is thought to be marked by a restoration of left-hemisphere dominance for language-related processing) is likely critical for the successful long-term recovery of language functions [12, 15–20]. Thus, while the upregulation of right-hemisphere responses during language task performance might reflect a form of compensatory reorganization, it is likely less effective than the reinstatement of left-hemisphere processing for accomplishing language task performance [13, 16, 20–23].

Studies investigating how changes in cortical function relate to language recovery following stroke provide strong evidence indicating that the preservation and/or restoration of language-related processing in the residual left inferior frontal gyrus (IFG), a region that has been strongly implicated in various language processes such as word processing and word generation [24–26], is strongly related to the recovery of language functions in both the acute and chronic stages of recovery. For example, adult patients with acute injury who show preserved dominance of the residual left IFG for language task performance have less severe language impairments than patients who depend on the compensatory recruitment of the right IFG to accomplish the same task, indicating that the preservation of language-related processing in this region after LMCA stroke is an important factor in determining initial aphasia severity [21, 22]. In addition, adult patients that receive early poststroke aphasia rehabilitation show enhanced language-related responses in the residual left IFG compared to patients that do not receive early rehabilitation, and the magnitude of treatment-related increases in left IFG responses during language task performance is correlated with improvements in language function after treatment [23]. Similarly, increases in the left-lateralization of IFG activity related to language task performance from early to chronic recovery phases correlate with improvements in naming ability in adult patients with poststroke aphasia [17], and the level of language-related activity in left frontal areas correlates with improvements in naming ability subsequent to behavioral treatments in chronic patients [27]. The development of treatments that can facilitate the restoration of language-related processing in residual frontal language areas may, therefore, be an important step in improving both spontaneous and treatment-induced recovery in patients with poststroke aphasia.

Techniques such as transcranial magnetic stimulation (TMS) enable the noninvasive manipulation of cortical excitability in specific parts of cortex and may provide a means for facilitating beneficial cortical plasticity in patients with poststroke aphasia [28, 29]. Experimental interventions utilizing these techniques typically attempt to induce changes in cortical function that mirror those observed in successfully recovered patients by transiently enhancing the excitability of residual left-hemisphere language areas or suppressing responses in their right-hemisphere homologues [29–31]. High-frequency TMS stimulation protocols (e.g., >5 Hz) such as intermittent theta burst stimulation (iTBS) are delivered in short intervals to produce a rapid facilitation of synaptic transmission in the stimulated cortex that can persist for over an hour after the initial stimulation session [32]. In addition to facilitating changes in local synaptic transmission and evoked potential amplitude, iTBS may also alter the temporal characteristics of ongoing oscillatory activity, suggesting that it may lead to changes in ongoing neural dynamics at larger spatial scales that reflect changes in the functional organization of distributed functional networks [33].

Excitatory stimulation protocols are typically applied to the residual left IFG in order to facilitate language-related processing [34–36]. In contrast, low-frequency stimulation protocols (e.g., <1 Hz) that are delivered in continuous trains for longer periods of time have predominantly inhibitory effects on synaptic transmission and are typically applied to the right IFG in order to reduce contralateral compensation and/or interference during language-related processing [36–40]. Studies investigating the efficacy of these paradigms for restoring language function after stroke have provided consistent evidence for improvements in language function subsequent to stimulation [34, 36–38]. Studies assessing the general effects of excitatory [34] and inhibitory [40] stimulation paradigms on neuroimaging measures of language-related responses in aphasic patients suggest that improvements in language function are accompanied by changes in the responses of both the residual left-hemisphere language network and homologous areas in the right hemisphere, although research in this area remains limited.

A previous behavioral and functional MRI (fMRI) study conducted by our laboratory found that after 10 sessions of iTBS applied to residual language-responsive left frontal cortex identified with a semantic decision/tone decision task, patients with chronic poststroke aphasia showed significant improvements in word generation as well as changes in fMRI responses during a semantic decision task that included a significant leftward shift in the lateralization of activity in the IFG [34]. In addition, a previous analysis of concurrently collected diffusion tensor imaging (DTI) data from the same patients found evidence for changes in white matter integrity in multiple regions including the left IFG following iTBS treatment [41]. However, a major limitation of our previous fMRI study is that it was restricted to changes in activation associated with the same task that was used to define language-responsive cortex for targeting with iTBS [34], and this limits inferences regarding whether or not similar effects might be observed for activation during other language tasks. Our previous fMRI analysis was also limited in that it only assessed changes in fMRI measures of activation, and it is increasingly recognized that the characterization of changes in measures of interregional connectivity is important for developing a full understanding of how changes in interregional interactions relate to the recovery of function after stroke [42, 43]. In addition, our previous structural analysis was restricted to investigating changes in white matter integrity after iTBS, although changes in cortical grey matter morphology might also be expected since excitatory TMS protocols have been found to result in measureable changes in cortical grey matter volume after as little as 5 days of treatment in individuals without stroke [44].

Here, we first analyzed the pre-/postintervention fMRI data to assess whether or not iTBS might have similar effects on fMRI responses elicited by a covert verb generation (VG) paradigm that was not used to define iTBS targets. Notably, while both the semantic decision paradigm used in our previous study and the VG paradigm used in the current study reliably evoke strong responses in the left IFG in healthy individuals [45] and in patients with poststroke aphasia [46], they target different functional domains (word comprehension versus word generation) and there is typically little overlap between the activations attained with these tasks beyond the left inferior frontal cortex [46]. We hypothesized that if iTBS has a general facilitatory effect on language-related processing in the residual left IFG (i.e., by modulating synaptic transmission to facilitate communication with other language-relevant areas or to suppress interference from language-irrelevant interactions with other areas), then patients should show increased responses in the left IFG during covert verb generation after treatment with iTBS and increased left-lateralization of IFG activity associated with covert verb generation. Because language lateralization in frontal cortex is associated with both higher levels of activity in the left hemisphere and lower levels of activity in the right hemisphere for language-related versus non-language-related tasks [47] and because our previous study also found evidence for decreased activity in the right IFG related to semantic decisions following iTBS [34], we expected that patients would also show reductions in right IFG activity during covert verb generation after iTBS. In addition, because studies of healthy individuals indicate that the presence of left-lateralized IFG activation during language tasks may be in part due to a task-dependent reduction in connectivity between left and right IFG [48] and because we are unaware of any studies that have investigated changes in functional MRI measures of connectivity in patients with poststroke aphasia subsequent to iTBS treatment, we also investigated whether or not interhemispheric connectivity between right and left IFG during covert verb generation was affected by iTBS. Finally, in order to fully characterize the structural effects of iTBS in these patients, we also tested whether or not patients showed changes in regional grey matter volume after iTBS treatment.

## 2. Materials and Methods

### 2.1. Patient Demographics and Language Testing

Eight prospectively identified patients (4 females; mean age = 54.4, SD = 12.7) with chronic aphasia resulting from LMCA stroke were recruited as described previously [34, 41]. The mean time since stroke for all patients included was 5.25 years (SD = 3.62). Aphasia types were determined by a linguistics expert following language testing. Four patients presented with anomic aphasias; of these subjects, two also presented with dysarthria and one also presented with conduction aphasia. The remaining four patients all presented with nonfluent Broca's type aphasias. Aphasia diagnoses and lesion characteristics are shown for each patient in Table 1. None of the patients had contraindications to MRI scanning, none had history of seizures, and all were right-handed prior to the stroke. The study was approved by the University of Cincinnati, Cincinnati Children's Hospital Medical Center, and University of Alabama at Birmingham Institutional Review Boards and adhered to the Declaration of Helsinki regarding human subject research. Each patient provided signed informed consent prior to inclusion in the study. Neuropsychological measures of language function were acquired before and after iTBS treatment as described in previous publications [34, 41]. Briefly, naming and word-finding abilities were evaluated using the Boston Naming Test (BNT) [49], receptive vocabulary was evaluated using the Peabody Picture Vocabulary Test (PPVT) [50], verbal fluency was evaluated using the Semantic Fluency Test (SFT) [51] and Controlled Oral Word Association Test (COWAT) [52], and comprehension was evaluated using the Complex Ideation Subtest of the Boston Diagnostic Aphasia Examination (BDAE CompId) [53]. Patients also completed the min-Communicative Abilities Log in order to provide subjective measurements of progress in verbal communication [54]. Pre- and posttreatment testing used different versions of the assessments in order to reduce the potential for learning-related effects.

### 2.2. Intermittent Theta Burst Stimulation Protocol

**Figure 1:**
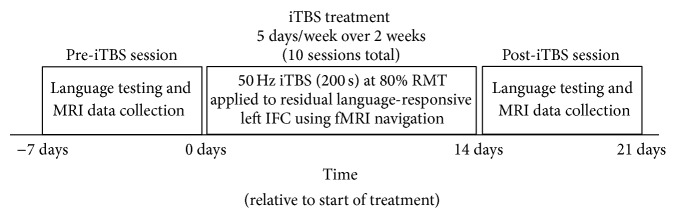
Experimental timeline. Patients underwent language testing and MRI scans during the week prior to treatment. Patients then received one session of iTBS each on weekday over a two-week period and underwent language testing and MRI scans again during the week following treatment.

Detailed descriptions of all iTBS and neuronavigation protocols performed on these patients can be found in our previous publication [34]. Briefly, all patients received iTBS to residual language-responsive cortex in or near the left IFG as identified using an fMRI semantic decision/tone decision language localizer task described in our previous publication [34]. Stimulation intensities used for each patient were set at 80% of the active motor threshold obtained from stimulation of the right motor cortex. Stimulation sessions occurred each day for five consecutive weekdays over the course of two weeks, resulting in a total of 10 stimulation sessions. Each session consisted of 600 total pulses, with three pulses at 50 Hz given every 200 milliseconds in 2-second trains at 10-second intervals over a 200-second period. fMRI-guided neuronavigation using BrainSight2 (Rogue Research Inc., Montreal Canada) enabled the targeting of residual language-responsive cortex in the left frontal lobe near the IFG (frontal targets were used for 7 patients; language-responsive cortex in the left temporal lobe was targeted for one patient; see Figure  1 in [34]) that was identified using the fMRI localizer task, and allowed for reliable and precise localization of the same location at each session. A schematic illustrating the experimental timeline is shown in Figure 1.

### 2.3. MRI Data Acquisition

MRI data were acquired before and after the treatment sessions. The functional and anatomical MRI data presented in this study were acquired using a Varian 4 Tesla Unity INOVA whole body MRI/MRS scanner (Varian, Inc., Palo Alto, CA). For each patient, a high-resolution T1-weighted 3D-MDEFT (Modified Driven Equilibrium Fourier Transform) anatomical volume (scan parameters: repetition time/echo time = 13.1/6 ms, field of view = 25.6 × 19.2 × 19.2 cm, flip angle = 22°, and voxel dimensions = 1 × 1 × 1 mm) and T2^*∗*^-weighted blood oxygen-level dependent (BOLD) volumes (scan parameters: repetition time/echo time = 3000/30 ms, FOV = 25.6 × 25.6 cm, matrix = 64 × 64 pixels, number of slices = 30, slice thickness = 4 mm, and flip angle = 75°) were obtained at both pretreatment and posttreatment sessions.

FMRI data were collected while patients performed an alternating block-design covert verb generation (VG) task that consisted of alternating 30 s blocks of an active condition involving silent verb generation in response to binaurally presented nouns and a control condition involving bilateral sequential finger tapping (FT) in response to a frequency modulated tone centered on 400 Hz that was modulated by 25% every 5 s. This task was chosen because previous studies indicate that it reliably produces left-lateralized activation patterns [45] and because it has excellent test-retest (across time points) reliability for evoked activity patterns in patients with aphasia due to LMCA stroke [46]. The control condition served to control for the auditory stimulation during the noun presentation in the active condition and to distract patients from continuing to generate verbs outside of the active condition blocks while maintaining a task state. Each condition was performed 7 times. Each patient's understanding of and ability to perform the task were assessed prior to scanning by having the patients perform the task outside of the scanner. Patients had to be able to generate at least one verb in response to each noun prior to proceeding to scanning. Following each scan session, patients performed a forced-choice recognition test involving the nouns that were presented during the covert verb generation task, and the percentage of correctly remembered nouns was utilized as an indirect measurement of task performance.

### 2.4. MRI Data Preprocessing

All MRI data were preprocessed using MATLAB scripts implementing functions from the most recent release version of Statistical Parametric Mapping (SPM12, Wellcome Department of Cognitive Neurology, London, UK) running in MATLAB r2014B (The MathWorks Inc., Natick, MA). All statistical analyses were performed using statistical functions provided in MATLAB.

Functional MRI data from the baseline and follow-up scans were slice-time corrected, realigned and resliced, and coregistered to the structural image obtained during the same scan. Deformation fields containing the deformation differences between across-session average anatomical volume and the anatomical scan from each session were used to warp the coregistered functional volumes to the across-session average anatomical volume. The average anatomical scan was then normalized to Montreal Neurological Institute (MNI) template using unified normalization-segmentation as implemented in the New Segment tool in SPM. The deformation parameters obtained from the warping of the anatomical volume were used to normalize the functional volumes to MNI template space. The functional volumes were resampled to 2 × 2 × 2 millimeter isometric voxels and spatially smoothed with a 6-millimeter full-width half maximum (FWHM) Gaussian kernel. Functional volumes in which participants moved more than 0.5 mm in one frame (3 s) were replaced with a volume interpolated from adjacent time points. Volumes were to be rejected if they contained >3 mm of motion, but no volumes met criteria for rejection.

**Figure 2:**
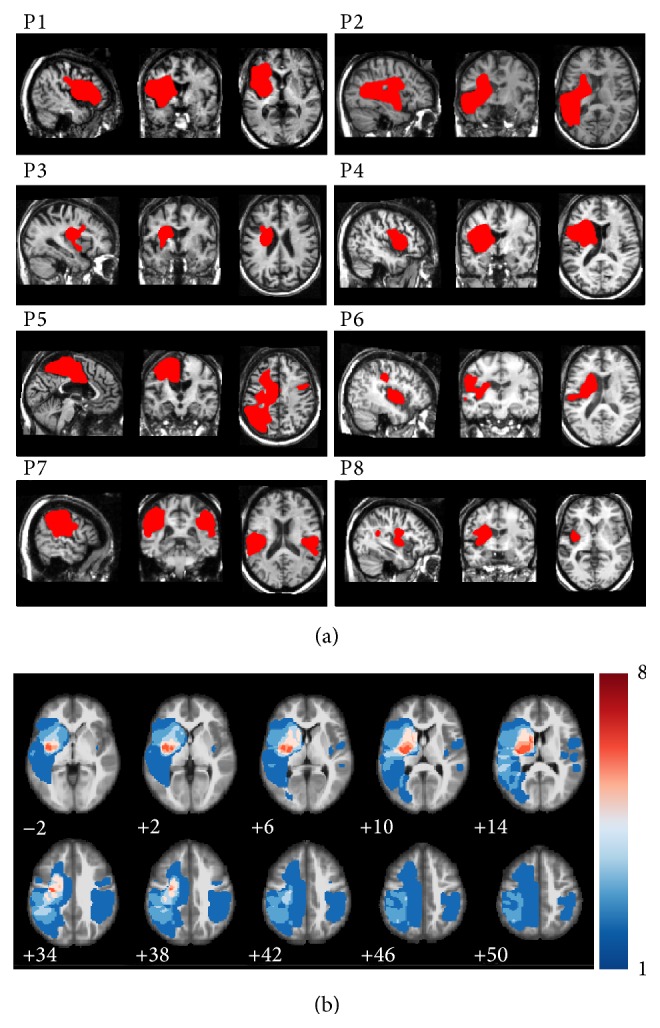
(a) Representative images from the normalized T1-weighted anatomical scans are shown for each patient; lesion delineations are shown in red. (b) A lesion frequency overlay for all 8 participants shown as a color-map overlaid on a template anatomical image. Colors represent the number of patients with a lesion at each voxel as indicated by the color bar.

Individual patient lesion delineations were created from the pre-iTBS anatomical scans using an automated voxel-based Bayesian classification algorithm developed by our lab and implemented in the lesion_gnb toolbox for SPM12 [55]. The resulting lesion delineations were used to create a group-level lesion frequency map. The group-level lesion frequency map is provided in Figure 2 and illustrates the number of patients with lesioning at each voxel. The greatest across-patient lesion overlap was observed in the left insula, left putamen, and left precentral gyrus (Figure 2).

Anatomical data utilized in the voxel-based morphometry (VBM) analysis were preprocessed according to a recently described longitudinal preprocessing pipeline for VBM analyses [56]. First, probabilistic tissue segmentation implementing the New Segment + DARTEL (diffeomorphic automatic registration through exponentiated lie algebra) approach with an additional tissue prior (mean of white matter and CSF tissue probability maps) and medium bias regularization was used to obtain DARTEL-compatible tissue probabilistic maps (TPMs) encoding the grey matter (GM) and white matter (WM) probabilities for each voxel. The additional tissue prior and medium bias regularization were used since this has been shown to improve template-space normalization using the New Segment + DARTEL approach [57]. Next, patient-specific anatomical templates were created with DARTEL using the GM and WM tissue maps from the baseline and follow-up scans. For each patient, the baseline and follow-up TPMs were then warped to the subject-specific templates and modulated using the Jacobian determinant of the transformation to increase sensitivity to absolute differences in GM volume [58, 59]. The creation of the patient-specific templates was performed in order to enable more precise between-session spatial alignment of TPMs [56]. A group template was then created by nonlinearly registering all of the patient-specific templates simultaneously using DARTEL. The modulated/warped GM and WM TPMs obtained from each patient were then nonlinearly normalized to the population template and modulated with the Jacobian determinant of the transformation. Finally, the population template was then registered to MNI space using an affine transformation, and each TPM was then coregistered to MNI space using an identical transformation and smoothed using an 8 mm FWHM Gaussian kernel.

### 2.5. Functional MRI Data Analyses

Functional MRI activity related to the covert verb generation task was quantified by contrasting the active condition blocks (VG) against the control condition blocks (FT). For each patient, the fMRI data were fit to a general linear model (GLM) [60] where each active block was modeled as a boxcar regressor convolved with a canonical hemodynamic response function. To account for temporal variability in the hemodynamic response, time and dispersion derivatives were modeled as basis functions in the first-level analyses [61]. Single-patient statistical maps containing contrast estimates quantifying differences between the active and control conditions were computed for both pre- and post-iTBS scans. Statistical comparisons of the contrast estimate maps were used to evaluate changes in activation between the pre- and post-iTBS sessions.

FMRI data were first analyzed using a region of interest (ROI) approach in order to directly test our hypotheses regarding changes in IFG activity and connectivity between pre- and post-iTBS sessions. ROI masks were created using the marsbar toolbox for SPM (http://marsbar.sourceforge.net/), and ROIs in the left and right IFG were defined using peak activation coordinates obtained from an independent analysis of activation related to covert verb generation in healthy individuals [45]. 8 mm radius spherical ROIs were centered on voxels in the left IFG (MNI coordinates: *x* = −50, *y* = 16, and *z* = 16) and on mirrored coordinates in the right IFG (MNI coordinates: *x* = 50, *y* = 16, and *z* = 16). Only two patients (P1 and P4) had overlap between the left IFG ROI and their lesion delineation. P1's lesion encompassed nearly the entire left lateral prefrontal cortex and the left IFG ROI fell directly onto the perilesional rim, resulting in 100% lesion overlap with the left IFG ROI. P4's lesion was primarily localized to the left ventral IFG and the ROI overlapped by 60.2% with the perilesional rim. Nonetheless, for both patients, the 1st principal component of the signal extracted from the ROI showed phasic responses consistent with the design of the task. While anatomical overlap was noted, functional analyses indicated BOLD signal changes aligned with the box-car function of the fMRI task design indicating that the fMRI responses reflected the responses of perilesional cortex rather than CSF. Additionally, signal from the left IFG ROI was more strongly correlated with signal from the right IFG ROI than with the CSF signal, and this was only marginally influenced by partialling out variability accounted for by the CSF signal (Supplemental S1) (see Supplementary Material available online at http://dx.doi.org/10.1155/2016/4796906). Nonetheless, control analyses indicated that excluding these patients did not substantially change the statistical significance of results for any analysis involving the left IFG ROI, indicating that the presence of lesion-ROI overlaps for these patients did not strongly influence the outcome of the ROI analyses.

To test our hypothesis about whether patients showed changes in fMRI activity during covert verb generation in the left and right IFG ROIs after iTBS, we extracted the mean parameter estimates for the active versus control condition contrast and compared the estimates obtained for each ROI between the pre- and post-iTBS sessions. This analysis provided information about the mean level of activity in the IFG ROIs for each session, with positive values indicating stronger activation during the active condition and negative values indicating stronger activation during the control condition. To test our hypothesis that patients would show more strongly left-lateralized IFG activation following iTBS, laterality index (LI) analyses were performed to quantify the lateralization of activity related to covert verb generation. For each patient, changes in LI were evaluated using an adaptive threshold determination approach [62]. LI values range from −1 (complete right-lateralization) to 1 (complete left-lateralization), and LIs for each session were calculated according to the formula shown in

(1) $$\begin{array}{c} LI=\frac{\left(\sum {activation}_{left}/mwf\right)-\sum {activation}_{right}}{\left(\sum {activation}_{left}/mwf\right)+\sum {activation}_{right}}. \end{array}$$

Using adaptive threshold determination, the term activation is defined as the values of voxels with intensities that are greater than the within-ROI average intensity for the contrast of interest. This method was chosen because it has been shown to provide reliable LI estimates that are more robust against interindividual variability in signal-to-noise ratio than approaches that employ arbitrary/fixed cut-off thresholds that are applied to all subjects (e.g., corrected *P* value thresholds); this method does not substantially increase susceptibility to false positives [62]. The mask weighting factor (mwf term in (1)) is used to adjust the LI estimates to account for differences in the volume of each ROI and is defined by the proportion of the volumes of the left- and right-hemisphere ROIs [62].

To test our hypothesis regarding the effects of iTBS on interhemispheric connectivity, we conducted a generalized psychophysiological interaction (gPPI) analysis using the gPPI toolbox for SPM [63]. gPPI enables the modeling of context-specific changes in the relationship of activity in one brain region, referred to as a seed region, to activity in other brain regions by including a term specifying an interaction effect between the seed region time series and the task time series in each first-level GLM [64]. gPPI effects are interpreted as changes in interregional connectivity which are driven by psychological states related to factors such as the task being performed [63, 65], making gPPI an appropriate tool for testing our hypothesis that iTBS would lead to changes in interhemispheric connectivity during covert verb generation. For each patient, the first principal component of the BOLD time series from each scan was extracted from the right IFG ROI and entered as a seed time series for the gPPI analysis. The right IFG ROI, rather than the left IFG ROI, was chosen in order to reduce potential confounds in the extracted time series related to lesion proximity since two patients showed substantial lesion overlap with the left IFG ROI. Cerebrospinal fluid (CSF) and WM signals were also included as nuisance variables in the gPPI model in order to reduce the influence of nonneural signals on estimates of task-dependent connectivity [66]. For each patient, gPPI estimates quantifying the level of condition-dependent connectivity from right to left IFG during each session were then extracted from the gPPI model using the marsbar tool for SPM and compared from pre- to post-iTBS sessions.

All between-session comparisons were tested for statistical significance using two-tailed dependent samples *t*-tests. Correlations between changes in fMRI measures of IFG function and behavioral measures were assessed using linear correlation analyses. Multiple-comparisons correction for all ROI-driven comparisons between pre-iTBS and post-iTBS scans was performed using the Benjamini-Hochberg procedure to control the false-discovery rate (FDR) at 0.05 [67, 68], and all associated *P* values presented are FDR-adjusted. Exploratory whole-brain GLM and gPPI analyses were also performed in order to provide a more thorough characterization of functional MRI measures of activity and right IFG connectivity related to the VG task at the pre-iTBS and post-iTBS sessions. Statistical tests for these analyses were performed using dependent samples *t*-contrasts. Exploratory and ad hoc partial correlational analyses were performed to further characterize the data and are presented with uncorrected *P* values.

### 2.6. Voxel-Based Morphometry Analyses

VBM is a technique that allows for the measurement of grey matter (GM) volume in T1-weighted MRI data [58, 69]. Here, we used VBM to address the question of whether or not patients showed changes in GM volume after iTBS treatment. The DARTEL-processed subject-level grey matter maps from baseline and follow-up scans were entered into a dependent samples *t*-contrast that also included each patient's lesion volume as a nuisance covariate. Changes in GM volume were assessed at the whole-brain level using dependent samples *t*-contrasts.

## 3. Results

### 3.1. Behavioral Results

Analyses evaluating performance on the out-of-scanner forced-choice noun recognition task revealed good performance for both pre-iTBS (mean % correct = 94.13; SEM = 1.63) and post-iTBS (mean % correct = 95.13; SEM = 2.05) sessions. Dependent samples *t*-test comparing pre-iTBS and post-iTBS evaluations did not reveal a significant change in noun recognition performance (*t*_7_ = 0.415, *P* = 0.69). These results are consistent with previous studies that have reported good out-of-scanner noun recognition performance on this task in patients with poststroke aphasia [46, 70].

The effects of iTBS on neuropsychological measures of language function have been previously reported [34, 41] and will briefly be described here to provide details relevant to the current study. Our previous analysis revealed that patients showed a statistically significant (at *P* < 0.05) improvement on the Semantic Fluency Test, statistically nonsignificant (at *P* < 0.05) improvements in performance on the Boston Naming Test (Boston Diagnostic Aphasia Examination, Peabody Picture Vocabulary Test, and the Communicative Abilities Log), and a statistically nonsignificant (at *P* < 0.05) decrease in performance on the Controlled Oral Word Association Test (see Table  1 in [34]). Since statistically significant improvements were only observed for performance on the Semantic Fluency Test, exploratory analyses investigating the relationship between changes in functional MRI measures of IFG activation/connectivity and the behavioral effects of iTBS were restricted to this test. While ideally behavioral correlations would have been performed on the out-of-scanner noun recognition task, the miniscule change between sessions and globally good performance precluded this approach. Importantly, the Semantic Fluency Test, like the covert verb generation task, required patients to generate words in response to a prompt. For the Semantic Fluency Test, patients generated as many words as they could think of that were congruent with category prompt (e.g., animals) with a 1-minute time limit, and performance on the Semantic Fluency test was measured by the number of congruent words produced within the 1-minute time limit. Thus, whereas the covert verb generation task required patients to silently generate verbs in response to presented nouns, the Semantic Fluency Test required patients to generate words in response to a given category.

### 3.2. Functional MRI Results

**Figure 3:**
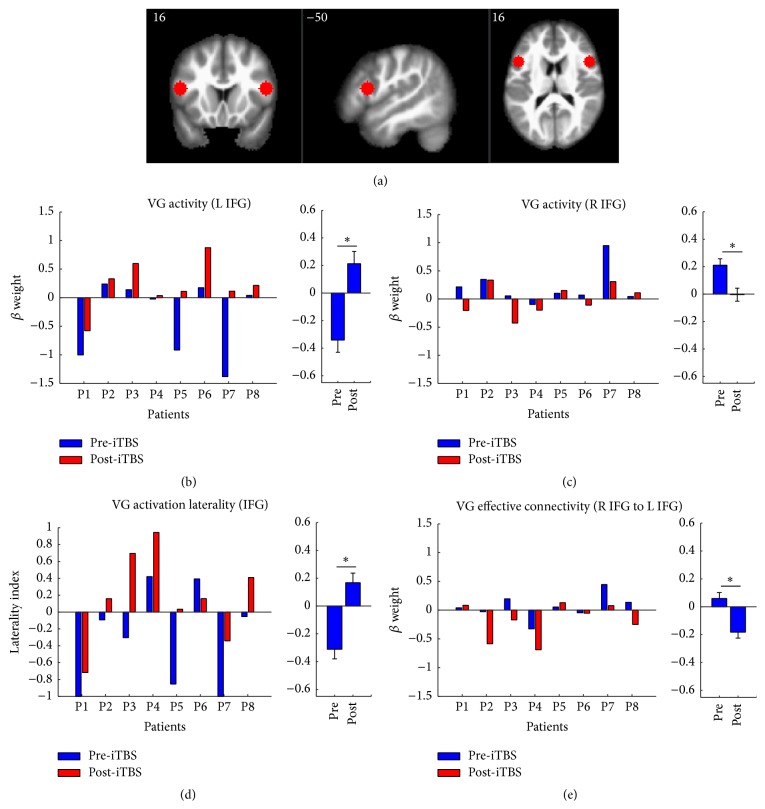
Results from ROI analyses. The left and right IFG ROIs are shown overlaid on slices from an anatomical template brain. The estimates of left (b) and right (c) IFG activation magnitudes, laterality indices (d), and effective connectivity from right to left IFG (e) are shown for the pre-iTBS (blue) and post-iTBS (red) sessions of each patient. For each plot in (b–e), bar graphs are shown on the right side that illustrate the mean and within-subjects standard error of the effect at pre-iTBS and post-iTBS sessions.  ^*∗*^Significant at FDR *P* = 0.05.

To test our hypotheses regarding the effects of iTBS on the magnitudes of activity in left and right IFG during covert verb generation, we compared activation magnitudes at each ROI between pre- and post-iTBS sessions. It is worth noting that the ROIs used for these analyses were chosen* a priori *in order to avoid the introduction of bias by defining ROIs based on the GLM results. Our analyses revealed increased activation magnitudes in the left IFG (*t*_7_ = 3.32; FDR *P* = 0.02; mean change = 0.54, SEM = 0.18) and decreased activation magnitudes in right IFG (*t*_7_ = −2.3; FDR *P* = 0.05; mean change = −0.22, SEM = 0.09) related to covert verb generation after iTBS treatment. Left and right IFG activation magnitudes for each patient are shown in Figures 3(b) and 3(c). On average, patients showed lower levels of activity in left IFG and higher levels of activity in right IFG during covert verb generation compared to finger tapping pre-iTBS. In contrast, patients showed higher levels of activity in left IFG and similar levels of activity in right IFG during covert verb generation compared to finger tapping post-iTBS. Accordingly, results from the LI analysis indicated that overall IFG responses during covert verb generation were more strongly left-lateralized after iTBS treatment (*t*_7_ = 3.46, FDR *P* = 0.02; mean change = 0.48, SEM = 0.14). LI estimates for each patient at pre-iTBS and post-iTBS sessions are shown in Figure 3(d). On average, patients showed right-lateralized activation patterns in IFG pre-iTBS. In contrast, patients showed left-lateralized activation patterns in IFG post-iTBS.

**Figure 4:**
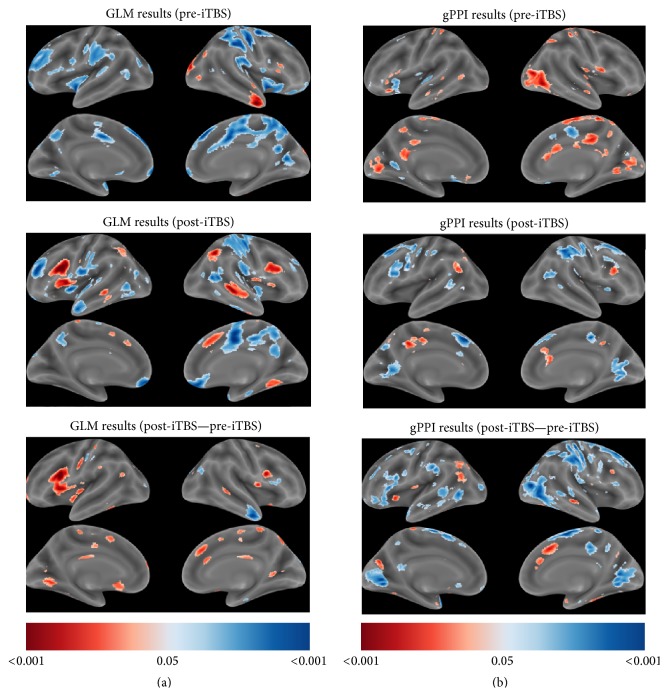
Whole-brain statistical maps for GLM and gPPI analyses. (a) Statistical parametric maps (SPMs) illustrating whole-brain activation for the VG > FT (red) and VG < FT (blue) contrasts from the pre-iTBS (top) and post-iTBS (middle) scan sessions are shown to illustrate overall activation patterns for each scan session (left, middle). An SPM illustrating changes in VG activation is also shown (bottom). (b) SPMs illustrating whole-brain gPPI results for the R IFG seed region for the VG > FT (red) and VG < FT (blue) contrasts from the pre-iTBS (top) and post-iTBS (bottom) scan sessions are shown to illustrate overall task-dependent connectivity patterns for each scan session. An SPM illustrating changes in task-dependent connectivity is also shown (bottom). Color bar values for all SPMs indicate uncorrected *P* values ranging from 0.05 to <0.001.

To allow for a more thorough characterization of the data, whole-brain GLM analyses were also performed. Although no regions showed significant effects at a whole-brain FDR-corrected threshold of 0.05, the uncorrected statistical maps provide evidence for increased responses related to covert verb generation in left-hemisphere frontal, temporal, and parietal regions after iTBS (Figure 4(a)). While no regions showed changes that were significant after multiple-comparisons correction, the most reliable (voxelwise *P* < 0.001, uncorrected) increases in activity were observed in the left IFG pars opercularis (peak MNI coordinate: −40, 14, 10; 171 voxel clusters), the right thalamus (peak MNI coordinate: 10, −14, 20; 64 voxel clusters), and the right cerebellum VI (peak MNI coordinate: 30, −62, −30; 3 voxel clusters), and the most reliable (voxelwise *P* < 0.001, uncorrected) decreases in activity were observed in the right cerebellum crus 2 (peak MNI coordinate: 48, −52, −42; 12 voxel clusters), the right cerebellum VIII (peak MNI coordinate: 34, −44, −40; 6 voxel clusters), and the right inferior temporal gyrus (peak MNI coordinate: 54, −6, −30; 4 voxel clusters).

To test our hypothesis regarding the effects of iTBS on effective connectivity between the right and left IFG, we compared gPPI estimates between the right IFG seed region and the left IFG target region between pre- and post-iTBS sessions. It is important to note that gPPI estimates reflect the magnitude of condition-dependent changes in the relationship between activity in the seed region and activity in the target region [63]. Thus, the connectivity estimates for each session quantify how the relationship between responses in the right IFG and responses in the left IFG differed between conditions. These analyses revealed that compared to the pre-iTBS session, patients showed reductions in gPPI estimates between the right IFG seed region and the left IFG target region for the active condition contrast at the post-iTBS session (*t*_7_ = −2.97; FDR *P* = 0.03; mean change = −0.24, SE = 0.09). The gPPI estimates for the active condition contrast for each patient are shown in Figure 3(e).

Since the gPPI measurement quantifies differences in the relationship between activity in the seed region (R IFG) and the target region (L IFG) that are moderated by task condition (VG-FT), a reduction in the gPPI estimate between right IFG and left IFG would indicate that the effect of right IFG activity on left IFG activity for covert verb generation relative to finger tapping was reduced after iTBS. Patients showed a small but positive mean effect of covert verb generation on connectivity between the right IFG and left IFG pre-iTBS, indicating that right IFG activity was more positively associated with left IFG activity during covert verb generation than during finger tapping. In contrast, patients showed a negative mean effect of covert verb generation on connectivity between the right IFG and left IFG post-iTBS, indicating that right IFG activity was more negatively associated with left IFG activity during covert verb generation than during finger tapping. Thus, the direction of the effect of task condition on the relationship between right IFG activity and left IFG activity changed between pre-iTBS and post-iTBS sessions, with right IFG activity being more negatively associated with left IFG activity during covert verb generation than during finger tapping.

To allow for a more thorough characterization of the data, whole-brain gPPI analyses were also performed. Although no regions showed significant effects at a whole-brain FDR-corrected threshold of 0.05, the uncorrected statistical maps provide evidence for reduced connectivity between the right IFG and left-hemisphere frontal, temporal, and parietal regions after iTBS (Figure 4(b)). While no regions showed changes that were significant after multiple-comparisons correction, the most reliable (voxelwise *P* < 0.001, uncorrected) reductions in right IFG connectivity associated with the VG task were observed in the right middle temporal gyrus (peak MNI coordinate: 36, −74, 6; 65 voxel clusters), the right superior frontal gyrus (peak MNI coordinate: 16, −2, 60; 32 voxel clusters), the left IFG pars opercularis (peak MNI coordinate: −50, 8, 22; 8 voxel clusters), the right postcentral gyrus (peak MNI coordinate: 36, −32, 56; 7 voxel clusters), the left lingual gyrus (peak MNI coordinate: −14, −70, 0; 6 voxel clusters), the right cerebellum VI (peak MNI coordinate: 24, −60, −24; 4 voxel clusters), the left caudate (peak MNI coordinate: −12, −4, 10; 4 voxel clusters), and the left temporal pole (peak MNI coordinate: −32, 10, −30; 1 voxel cluster). Interestingly, comparable (voxelwise *P* < 0.001, uncorrected) increases in right IFG connectivity associated with the VG task were not observed after iTBS.

### 3.3. Exploratory Behavioral Correlation Results

Prior to assessing behavioral correlations with fMRI measures of IFG function, correlations between total lesion volume and each measure were assessed. This revealed moderate but nonsignificant correlations between total lesion volume and changes in L IFG activity (*r* = 0.5, *P* = 0.2) and changes in connectivity (*r* = 0.33, *P* = 0.43) and weak but nonsignificant correlations between total lesion volume and right IFG activity (*r* = 0.11, *P* = 0.79). Thus, partial linear correlations were computed to investigate the relationship between the changes in functional MRI measurements of IFG function during covert verb generation and changes in performance on the Semantic Fluency Test following iTBS that were not attributable to total lesion volume. These analyses did not reveal significant correlations between overall changes in LI and changes in performance (partial *r* = −0.03, *P* = 0.96), changes in the magnitude of left IFG activity and changes in performance (partial *r* = 0.16, *P* = 0.74), or changes in the effects of covert verb generation on interhemispheric connectivity and changes performance (partial *r* = 0.15, *P* = 0.76). These analyses did reveal a strong negative correlation between changes in the magnitude of right IFG activity and changes in performance (partial *r* = −0.82, *P* = 0.01), indicating that decreases in right IFG activity during covert verb generation between pre/post-iTBS sessions were associated with concurrent improvements on the Semantic Fluency Test.

### 3.4. Ad Hoc Functional MRI Analysis Results

To further explore the effects of iTBS on IFG function, additional analyses were performed on the fMRI data. Our* a priori* analyses indicated that iTBS was associated with changes in the responses of both the left IFG and right IFG during covert verb generation and also indicated that iTBS was associated with reduced connectivity from right IFG to left IFG during covert verb generation. The exploratory behavioral correlation analyses also revealed the somewhat surprising result that post-iTBS improvements in performance on the Semantic Fluency Test were most strongly related to reductions in the responses of right IFG during covert verb generation. These findings led us to question whether the relationship between post-iTBS changes in the responses of left and right IFG during covert verb generation showed a consistent pattern across patients. They also led us to question whether the effects of iTBS on the responses of left and right IFG during covert verb generation might relate to the pretreatment levels of effective connectivity from right IFG to left IFG during covert verb generation. It might be expected, for example, that preexisting interhemispheric dynamics might influence the effects of high-frequency iTBS on the function of left and right IFG. These questions were addressed using additional exploratory partial correlation analyses that, while not related to our initial hypotheses, were included to more fully characterize the data.

First, we addressed the question of whether or not changes in left IFG activity after iTBS were correlated with changes in right IFG activity after iTBS while controlling for total lesion volume. This revealed a nonsignificant negative correlation between post-iTBS changes in left and right IFG activity (partial *r* = −0.65, *P* = 0.12). Second, we addressed the question of whether or not the effects of iTBS on left and right IFG activation magnitudes were correlated with the effects of covert verb generation on interhemispheric connectivity prior to iTBS treatment. This revealed a positive correlation between pre-iTBS effects of verb generation on interhemispheric connectivity and changes in left IFG activation magnitude after iTBS treatment (partial *r* = 0.75, *P* = 0.05). A nonsignificant negative correlation was found between pre-iTBS connectivity and changes in right IFG activation magnitude after iTBS (partial *r* = −0.60, *P* = 0.15).

### 3.5. Voxel-Based Morphometry Analyses

**Figure 5:**
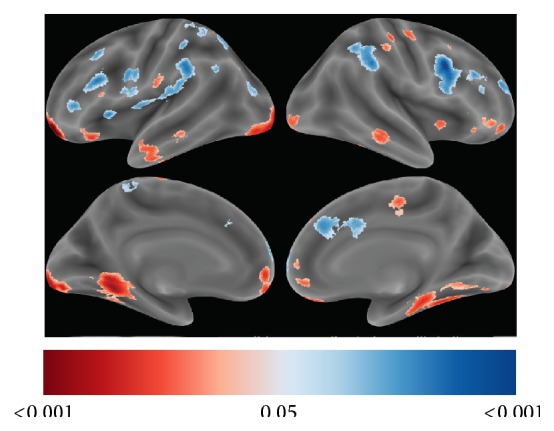
Whole-brain statistical maps VBM analyses. Statistical parametric maps (SPMs) illustrating increases (red) and decreases (blue) in GM volume following iTBS. Color bar values for all SPMs indicate uncorrected *P* values ranging from 0.05 to 0.001.

VBM was used to test our hypothesis that patients would show changes in GM volume between pre- and post-iTBS sessions. An initial analysis using a voxelwise FDR threshold of 0.05 did not reveal any effects of iTBS on GM volume. An evaluation of the unthresholded statistical maps (Figure 5) revealed that the most reliable (*P* < 0.001, uncorrected) increases in GM volume occurred in the left medial orbital gyrus (peak MNI coordinates: −22, 52, −14; 123 voxel clusters) and in the left lingual gyrus (peak MNI coordinates: −20, −94, −14), and the most reliable (*P* < 0.001, uncorrected) decreases in GM volume occurred in the right superior frontal gyrus (peak MNI coordinates: 18, 66, 12; 8 voxel clusters) and in the right IFG pars opercularis (peak MNI coordinates: 36, 6, 30; 6 voxel clusters).

## 4. Discussion

Growing evidence supports the use of techniques that utilize the noninvasive modulation of cortical excitability to improve language functions in patients poststroke aphasia [28, 29, 34, 37, 40, 71–73]. However, the development and optimization of future treatment protocols that harness the full potential of these techniques are limited by a rudimentary understanding of the changes in neural function that enable their therapeutic effects [74]. The present study provides insights into this issue by characterizing changes in cortical function and structure following 10 sessions of iTBS applied to residual language-responsive cortex in a group of chronic poststroke aphasia patients. Our results show evidence for changes in language task-related responses in both the stimulated and unstimulated hemispheres following iTBS treatment that are characterized by a general shift from right-lateralized to left-lateralized responses. Moreover, we show evidence for changes in the language task-related connectivity of right-hemisphere homologues of residual left-hemisphere language areas subsequent to iTBS treatment. These findings both replicate our previously reported observations of post-iTBS changes in left- and right-hemisphere responses related to semantic decisions [34] and extend them by demonstrating that similar changes in activation as well as additional changes in interhemispheric connectivity are observed during a covert verb generation task. Importantly, the current study utilized a language task (covert verb generation) that targets different aspects of language function from our previous study, which focused on semantic decisions, and that is independent of the stimulation targeting paradigm [34]. In addition, behavioral partial correlation analyses revealed that post-iTBS changes in the function of contralesional cortex showed a strong relationship to improvements in neuropsychological measures of language function after treatment that could not be explained by interindividual differences in lesion extents. Although we only found preliminary evidence for changes in regional grey matter volume following treatment with iTBS, our results nonetheless provide evidence of structural and functional neuroplasticity subsequent to a short-duration iTBS treatment in patients with poststroke aphasia. While preliminary, these results provide important insights into the changes in cortical function that enable improvements in language abilities following iTBS treatment.

The rerecruitment of residual left-hemisphere cortex for language processes is likely an important factor for the optimal long-term recovery of language functions following LMCA stroke [12, 14–17, 19, 21, 23]. Noninvasive techniques such as iTBS may facilitate the reintegration of residual cortex into cortical language networks by promoting beneficial neuroplasticity [29, 40]. While the mechanisms underlying the neuroplastic effects of iTBS in stroke patients are not fully understood, they likely involve multiple factors including modulations of gene expression, growth factor production, neurotransmitter release, and the facilitation of synaptic plasticity [74]. Our findings provide support for the conclusion that iTBS can induce plastic changes in the function of both the stimulated and unstimulated hemispheres in patients with chronic poststroke aphasia. Indeed, our ROI results suggest that the application of iTBS to residual language-responsive cortex in the left hemisphere has the potential to reduce contralesional compensation, increase residual left-hemisphere recruitment for language task performance, and alter task-dependent interhemispheric connectivity (Figure 3). While exploratory in nature, the results from our whole-brain analyses support these conclusions. Our GLM analysis found evidence for distinct patterns of left versus right-hemisphere activity during pre-iTBS and post-iTBS sessions, with left-hemisphere frontotemporal areas showing increased activity related to covert verb generation post-iTBS (Figure 4(a)). Similarly, our gPPI analysis found evidence for large-scale changes in the connectivity of right IFG during covert verb generation between pre-iTBS and post-iTBS sessions, with widespread reductions in right IFG connectivity being observed after iTBS (Figure 4(b)).

A speculative explanation for the observed effects is that the changes in left versus right IFG activity and connectivity during covert verb generation reflect the reinstatement of balanced inhibitory interactions between left and right IFC [13, 75]. Disproportionate influences of right-hemisphere homologues on left-hemisphere language areas have been previously documented in patients with aphasia resulting from LMCA stroke [76], and it is possible that heightened right IFG activation during language tasks reflects the release of transcallosal inhibitory outputs from left to right IFG following left-hemisphere damage [14, 75]. While not reaching our threshold for statistical significance, the results of our post hoc analyses suggested that increases in left IFG activation were related to decreases in right IFG activation regardless of lesion extent. This suggests that changes in right IFG activation depended in part on changes in the function of the left IFG, although this result is preliminary and should be interpreted as such. More highly powered analyses of larger samples are therefore necessary before conclusions about this relationship can be definitively drawn.

Additionally, results from our post hoc analyses indicated that the effects of iTBS on left and right IFG function had opposite relationships with the pre-iTBS strength of right IFG to left IFG connectivity during covert verb generation. While exploratory and requiring additional validation, these results have important implications, as they suggest that preexisting interhemispheric dynamics contribute to the effects of iTBS on the function of both the stimulated and unstimulated hemispheres. This interpretation is consistent with results from a recent study that indicated that the preservation of frontal white matter tracts, specifically the left arcuate fasciculus, explains substantial interindividual variability in behavioral improvements following cathodal TDCS applied to right IFG in patients with left IFG lesions [77]. Our results indicate that patients that showed stronger right to left IFG connectivity during covert verb generation at the pre-iTBS session also showed the most pronounced effects of iTBS on both left and right IFG activation magnitudes at the post-iTBS session. While future studies comparing connectivity between stroke patients in healthy controls are necessary to fully understand the implications of these findings, the general implications are that iTBS treatment can lead to increased left IFG activity/reduced right IFG activity and reduced right IFG to left IFG connectivity during language task performance and that the effects of high-frequency iTBS on both left and right IFG activation likely depend on the preexisting interhemispheric state prior to treatment.

Nevertheless, it is pertinent to address the question of how such large-scale changes in cortical function might result from the passive stimulation of residual left-hemisphere areas. After stroke, the loss of large-scale neural populations is thought to result in an acute breakdown of function in large-scale cortical networks that enable complex cognitive functions such as language [78] and attention [79]. This abrupt disruption of neural communication and regulation might be conceptualized as a large-scale perturbation of the brain's functional state that alters the trajectory of ongoing neural signaling [42]. During recovery, adaptive changes in the residual neural populations are thought to allow for the restoration of interregional communication and regulation, and it has been proposed that successful recovery of function after stroke may reflect the restoration of a near-normal functional state, whereas poor recovery may reflect ineffective reorganization that results in an aberrant functional state that is maladapted to generating normal cognitive/behavioral outputs [42]. Thus, it might be speculated that by passively stimulating the residual left-hemisphere cortex iTBS might induce changes in the state of local neural populations that facilitate the restoration of a closer-to-normal functional brain state. An expected outcome in such a scenario would be that following iTBS patients would show task/stimulus-evoked responses that more closely resemble those observed in healthy individuals. While preliminary, the observed changes in language-related responses and interhemispheric connectivity resemble the patterns of language-related responses [24, 47] and task-dependent connectivity [48] that are observed in healthy individuals with typical language function.

While the combination of iTBS with active language therapy might also be expected to lead to an enhancement of beneficial neuroplasticity, the expectation that passive stimulation can lead to changes in task-driven responses and connectivity is not unfounded. Indeed, the cortical/subcortical networks that underlie cognitive and behavioral functions such as language and attention maintain ongoing interregional signaling even when tasks are not being performed [80–83]. Importantly, disruptions in resting state cortical networks are observed in stroke patients [43], and the structure of residual resting state networks is also altered by treatment [84]. Thus, it might be expected that passive high-frequency stimulation of a residual but dysfunctional language network node might lead to the strengthening of synaptic connections with other language network nodes and that this may facilitate the eventual reintegration of the stimulated node to the residual language network. Such an effect would be consistent with the capabilities of iTBS to induce LTP-like changes in synaptic transmission that persist beyond the stimulation period [32] and with reports that these effects are paralleled by changes in the temporal coordination of large-scale, low-frequency oscillatory activity [33].

### 4.1. Limitations

The present study has limitations that must be acknowledged and considered in interpreting the results and in designing future studies. Primarily, the lack of a sham-stimulation group precludes the ability to make definitive statements about whether the observed effects are specific to iTBS treatment. It is worth noting that activation patterns during the covert verb generation task have been found to be remarkably consistent across time in patients with chronic poststroke aphasia [46], and the presence of reliable effects across chronic stroke patients in the present study indicates that the observed effects are not likely due to spontaneous changes in IFG function. Nonetheless, future studies that employ a sham-stimulation control are necessary to make definitive statements regarding the neuroplastic effects of iTBS in patients with chronic poststroke aphasia.

While sham-controlled studies investigating the effects of neurostimulation on language function in patients with chronic poststroke aphasia have consistently reported behavioral improvements that are specific to real stimulation [36, 71, 73, 85], most of these have used stimulation protocols involving the application of low-frequency rTMS to the unaffected right IFG. This does make our finding that behavioral improvements subsequent to high-frequency stimulation of left IFG negatively correlated with changes in right IFG activation particularly noteworthy, as this finding supports the use of paradigms such as these that aim to suppress dysfunctional activity in right IFG. While it is possible that both approaches may be manipulating similar mechanisms to achieve improvements in behavior, it is important for future studies to characterize the similarities and differences in the neuroplastic effects induced by each approach and to identify the mechanisms by which beneficial behavioral effects are achieved. However, since iTBS increases local cortical excitability and potentiates cortical evoked responses [32, 33], it might potentiate language-related responses in residual IFG or lead to changes in LI estimates even in the absence of beneficial neuroplastic effects. For example, in the absence of some preserved interactions between left and right IFG, high-frequency stimulation of left IFG (or low-frequency stimulation of right IFG) might lead to unilateral changes in activation during language tasks that could present as a transient overall shift in LI estimates. This might provide an explanation for the absence of a relationship between overall changes in LI and improvements in SFT in the current study and for the absent [71] or weak [40] relationships between changes in LI and behavioral improvements reported by other studies that have applied low-frequency rTMS to right IFG. Indeed, while measurements such as LI provide useful summary statistics, they face intrinsic limitations that likely limit their utility in assessing the specific effects of iTBS [86]. Thus, future studies investigating the effects of iTBS in this population should consider independently the changes in left versus right-hemisphere function in addition to assessing changes in summary statistics such as LI.

A second limitation to this study is the relatively small sample size. Although some would argue that this property makes the observed significant effects more compelling since the likelihood of finding significant-but-trivial effects increases with sample size, our limited sample likely also led to the obscuration of real effects due to relatively low power [87]. Indeed, in the current study, the detrimental effects of having a small sample size would most likely manifest as insufficient power to detect real effects, especially for the whole-brain GLM, gPPI, and VBM analyses at multiple-comparisons corrected thresholds. As we did not find any significant effects at corrected *P* value thresholds for the whole-brain analyses, the results from these analyses should be interpreted with caution. As such, we have refrained from drawing strong conclusions about the effects of iTBS on GM volume or on activity/connectivity beyond those examined with our ROI analyses.

While the whole-brain results are exploratory and thus should not be used to draw strong conclusions about the effects of iTBS, they do merit discussion. Regarding the observed effects of iTBS on regional GM volume, it would not be surprising if iTBS did have an effect on GM morphology in these patients, as detectable changes following rTMS have been reported after in as little as five days by studies investigating the effects of rTMS on cortical morphology in healthy individuals [44]. It is also worth noting that the previously reported effects of iTBS on white matter integrity in these patients were obtained using similarly lenient thresholds [41], and given the large variability in lesion etiologies for the patients in this study, it is thus perhaps not surprising that stronger effects were not observed here. While the direction and locations of some of the most reliable VBM effects (increased GM in left prefrontal areas/decreased GM in right IFG) are in line with our expected results, the current results do not provide basis for strong conclusions but do provide support for future investigations into these effects. Similarly, as noted earlier in the discussion, the whole-brain GLM and gPPI results do show effects consistent with larger-scale changes in the responses and interactions of the residual language network during covert verb generation. Thus, future studies with larger sample sizes are necessary to provide a full characterization of the effects of iTBS in this population.

In conclusion, we investigated the effects of iTBS applied to residual language-responsive cortex in the left hemisphere on MRI measurements of cortical function and structure in eight patients with chronic poststroke aphasia. We found that iTBS was associated with increased left-lateralization of IFG activity during covert verb generation. Changes in lateralization were characterized by increases in left IFG activation magnitudes and decreases in right IFG activation magnitudes that presented as an overall shift in the lateralization of IFG activity during covert verb generation. iTBS also led to reduced right to left IFG connectivity during covert verb generation, consistent with our interpretation that the effects of iTBS are related in part to changes in context-dependent interhemispheric interactions. Interestingly, our post hoc analyses suggest that the effects of iTBS on left and right IFG function were negatively correlated across patients (increased left IFG activity was associated with decreased right IFG activity), and the changes in left versus right IFG responses had opposite relationships to pre-iTBS levels of right IFG to left IFG connectivity during covert verb generation. These data provide insights into the neuroplastic changes associated with iTBS applied to residual left-hemisphere language areas in the treatment of chronic poststroke aphasia and provide support for future research in this area. Randomized, blinded, and sham-controlled studies in a larger sample of patients are necessary and are currently in progress (e.g., NCT01512264) in order to better clarify the neuroplastic effects of iTBS in this population.

## Supplementary Material

The Supplementary Material show raw BOLD time-series from each ROI for both patients with lesion-ROI overlaps in Supplementary Figure 1. The purpose of this figure is to illustrate that the time-series from the left IFG ROI, although it overlaps partially with the lesion, shows phasic responses that are similar to those observed for the right IFG ROI (where there is no lesion overlap). In addition, correlational analyses are provided to show that for both patients, the left IFG ROI time-series was more strongly correlated with the right IFG ROI than with the CSF time-series, and that the right-left IFG correlation was only marginally affected by removal of the CSF signal. These analyses demonstrate that the ROI-lesion overlap was unlikely to introduce artifactual effects into the left IFG signal.

## Figures and Tables

**Table 1:** Patient characteristics.

Patient	Aphasia diagnosis	Total lesion volume (voxels)
P1	Anomia, mild dysarthria	29,243
P2	Nonfluent (Broca-type)	32,744
P3	Anomia, mild dysarthria	1,436
P4	Anomia	20,195
P5	Nonfluent (Broca-type)	52,452
P6	Anomia, conduction	13,208
P7	Anomia	36,479
P8	Nonfluent (Broca-type)	7,269

## References

[1] Yarnell P., Monroe P., Sobel L. (1976). Aphasia outcome in stroke: a clinical neuroradiological correlation. *Stroke*.

[2] Pedersen P. M., Jørgensen H. S., Nakayama H., Raaschou H. O., Olsen T. S. (1995). Aphasia in acute stroke: incidence, determinants, and recovery. *Annals of Neurology*.

[3] Charidimou A., Kasselimis D., Varkanits M., Selai C., Potagas C., Evdokimidis I. (2014). Why is it difficult to predict language impairment and outcome in patients with aphasia after stroke?. *Journal of Clinical Neurology*.

[4] Bastian H. C. (1887). On different kinds of aphasia, with special reference to their classification and ultimate pathology. *British Medical Journal*.

[5] Mohr J. P., Pessin M. S., Finkelstein S., Funkenstein H. H., Duncan G. W., Davis K. R. (1978). Broca aphasia: pathologic and clinical. *Neurology*.

[6] Berker E. A., Berker A. H., Smith A. (1986). Translation of Broca's 1865 report. Localization of speech in the third left frontal convolution. *Archives of Neurology*.

[7] Dronkers N. F., Plaisant O., Iba-Zizen M. T., Cabanis E. A. (2007). Paul Broca's historic cases: high resolution MR imaging of the brains of Leborgne and Lelong. *Brain*.

[8] Krestel H., Annoni J.-M., Jagella C. (2013). White matter in aphasia: a historical review of the Dejerines' studies. *Brain and Language*.

[9] Saur D., Kreher B. W., Schnell S. (2008). Ventral and dorsal pathways for language. *Proceedings of the National Academy of Sciences of the United States of America*.

[10] Kümmerer D., Hartwigsen G., Kellmeyer P. (2013). Damage to ventral and dorsal language pathways in acute aphasia. *Brain*.

[11] Fridriksson J., Fillmore P., Guo D., Rorden C. (2015). Chronic Broca's Aphasia is caused by damage to Broca's and Wernicke's areas. *Cerebral Cortex*.

[12] Saur D., Lange R., Baumgaertner A. (2006). Dynamics of language reorganization after stroke. *Brain*.

[13] Heiss W.-D., Thiel A. (2006). A proposed regional hierarchy in recovery of post-stroke aphasia. *Brain and Language*.

[14] Turkeltaub P. E., Messing S., Norise C., Hamilton R. H. (2011). Are networks for residual language function and recovery consistent across aphasic patients?. *Neurology*.

[15] Karbe H., Thiel A., Weber-Luxenburger G., Herholz K., Kessler J., Heiss W.-D. (1998). Brain plasticity in poststroke aphasia: what is the contribution of the right hemisphere?. *Brain and Language*.

[16] Rosen H. J., Petersen S. E., Linenweber M. R. (2000). Neural correlates of recovery from aphasia after damage to left inferior frontal cortex. *Neurology*.

[17] van Oers C. A. M. M., Vink M., van Zandvoort M. J. E. (2010). Contribution of the left and right inferior frontal gyrus in recovery from aphasia. A functional MRI study in stroke patients with preserved hemodynamic responsiveness. *NeuroImage*.

[18] Fridriksson J. (2010). Preservation and modulation of specific left hemisphere regions is vital for treated recovery from anomia in stroke. *The Journal of Neuroscience*.

[19] Fridriksson J., Bonilha L., Baker J. M., Moser D., Rorden C. (2010). Activity in preserved left hemisphere regions predicts anomia severity in aphasia. *Cerebral Cortex*.

[20] Szaflarski J. P., Allendorfer J. B., Banks C., Vannest J., Holland S. K. (2013). Recovered vs. not-recovered from post-stroke aphasia: the contributions from the dominant and non-dominant hemispheres. *Restorative Neurology and Neuroscience*.

[21] Winhuisen L., Thiel A., Schumacher B. (2005). Role of the contralateral inferior frontal gyrus in recovery of language function in poststroke aphasia: a combined repetitive transcranial magnetic stimulation and positron emission tomography study. *Stroke*.

[22] Winhuisen L., Thiel A., Schumacher B. (2007). The right inferior frontal gyrus and poststroke aphasia: a follow-up investigation. *Stroke*.

[23] Mattioli F., Ambrosi C., Mascaro L. (2014). Early aphasia rehabilitation is associated with functional reactivation of the left inferior frontal gyrus: a pilot study. *Stroke*.

[24] Binder J. R., Frost J. A., Hammeke T. A., Cox R. W., Rao S. M., Prieto T. (1997). Human brain language areas identified by functional magnetic resonance imaging. *The Journal of Neuroscience*.

[25] Poldrack R. A., Wagner A. D., Prull M. W., Desmond J. E., Glover G. H., Gabrieli J. D. E. (1999). Functional specialization for semantic and phonological processing in the left inferior prefrontal cortex. *NeuroImage*.

[26] Costafreda S. G., Fu C. H. Y., Lee L., Everitt B., Brammer M. J., David A. S. (2006). A systematic review and quantitative appraisal of fMRI studies of verbal fluency: role of the left inferior frontal gyrus. *Human Brain Mapping*.

[27] Fridriksson J., Richardson J. D., Fillmore P., Cai B. (2012). Left hemisphere plasticity and aphasia recovery. *NeuroImage*.

[28] Naeser M. A., Martin P. I., Ho M. (2012). Transcranial magnetic stimulation and aphasia rehabilitation. *Archives of Physical Medicine and Rehabilitation*.

[29] Shah P. P., Szaflarski J. P., Allendorfer J., Hamilton R. H. (2013). Induction of neuroplasticity and recovery in post-stroke aphasia by non-invasive brain stimulation. *Frontiers in Human Neuroscience*.

[30] Raffin E., Siebner H. R. (2014). Transcranial brain stimulation to promote functional recovery after stroke. *Current Opinion in Neurology*.

[31] Di Pino G., Pellegrino G., Assenza G. (2014). Modulation of brain plasticity in stroke: a novel model for neurorehabilitation. *Nature Reviews Neurology*.

[32] Huang Y.-Z., Edwards M. J., Rounis E., Bhatia K. P., Rothwell J. C. (2005). Theta burst stimulation of the human motor cortex. *Neuron*.

[33] Papazachariadis O., Dante V., Verschure P. F. M. J., Del Giudice P., Ferraina S. (2014). iTBS-induced LTP-like plasticity parallels oscillatory activity changes in the primary sensory and motor areas of macaque monkeys. *PLoS ONE*.

[34] Szaflarski J. P., Vannest J., Wu S. W., DiFrancesco M. W., Banks C., Gilbert D. L. (2011). Excitatory repetitive transcranial magnetic stimulation induces improvements in chronic post-stroke aphasia. *Medical Science Monitor*.

[35] Holland R., Leff A. P., Josephs O. (2011). Speech facilitation by left inferior frontal cortex stimulation. *Current Biology*.

[36] Khedr E. M., Abo El-Fetoh N., Ali A. M. (2014). Dual-hemisphere repetitive transcranial magnetic stimulation for rehabilitation of poststroke aphasia: a randomized, double-blind clinical trial. *Neurorehabilitation and Neural Repair*.

[37] Naeser M. A., Martin P. I., Nicholas M. (2005). Improved picture naming in chronic aphasia after TMS to part of right Broca's area: an open-protocol study. *Brain and Language*.

[38] Barwood C. H. S., Murdoch B. E., Whelan B.-M. (2011). Improved language performance subsequent to low-frequency rTMS in patients with chronic non-fluent aphasia post-stroke. *European Journal of Neurology*.

[39] Kindler J., Schumacher R., Cazzoli D. (2012). Theta burst stimulation over the right Broca's homologue induces improvement of naming in aphasic patients. *Stroke*.

[40] Thiel A., Hartmann A., Rubi-Fessen I. (2013). Effects of noninvasive brain stimulation on language networks and recovery in early poststroke aphasia. *Stroke*.

[41] Allendorfer J. B., Storrs J. M., Szaflarski J. P. (2012). Changes in white matter integrity follow excitatory rTMS treatment of post-stroke aphasia. *Restorative Neurology and Neuroscience*.

[42] Carter A. R., Shulman G. L., Corbetta M. (2012). Why use a connectivity-based approach to study stroke and recovery of function?. *NeuroImage*.

[43] Rehme A. K., Grefkes C. (2013). Cerebral network disorders after stroke: evidence from imaging-based connectivity analyses of active and resting brain states in humans. *The Journal of Physiology*.

[44] May A., Hajak G., Gänßbauer S. (2007). Structural brain alterations following 5 days of intervention: dynamic aspects of neuroplasticity. *Cerebral Cortex*.

[45] Szaflarski J. P., Holland S. K., Jacola L. M., Lindsell C., Privitera M. D., Szaflarski M. (2008). Comprehensive presurgical functional MRI language evaluation in adult patients with epilepsy. *Epilepsy and Behavior*.

[46] Eaton K. P., Szaflarski J. P., Altaye M. (2008). Reliability of fMRI for studies of language in post-stroke aphasia subjects. *NeuroImage*.

[47] Seghier M. L., Kherif F., Josse G., Price C. J. (2011). Regional and hemispheric determinants of language laterality: implications for preoperative fMRI. *Human Brain Mapping*.

[48] Seghier M. L., Josse G., Leff A. P., Price C. J. (2011). Lateralization is predicted by reduced coupling from the left to right prefrontal cortex during semantic decisions on written words. *Cerebral Cortex*.

[49] Kaplan E., Goodglass H., Weintraub S., Segal O., van Loon-Vervoorn A. (2001). *Boston Naming Test*.

[50] Dunn D. M., Dunn L. M. (2007). *Peabody Picture Vocabulary Test*.

[51] Kozora E., Cullum C. M. (1995). Generative naming in normal aging: total output and qualitative changes using phonemic and semantic constraints. *Clinical Neuropsychologist*.

[52] Lezak M. D., Howieson D. B., Loring D. W., Hannay J. H., Fischer J. S. (1995). *Neuropsychological Assessment (3)*.

[53] Goodglass H., Kaplan E., Barresi B. (1972). *The Assessment of Aphasia and Related Disorders*.

[54] Pulvermüller F., Neininger B., Elbert T. (2001). Constraint-induced therapy of chronic aphasia after stroke. *Stroke*.

[55] Griffis J. C., Allendorfer J. B., Szaflarski J. P. (2016). Voxel-based Gaussian naïve Bayes classification of ischemic stroke lesions in individual T1-weighted MRI scans. *Journal of Neuroscience Methods*.

[56] Asami T., Bouix S., Whitford T. J., Shenton M. E., Salisbury D. F., Mccarley R. W. (2012). Longitudinal loss of gray matter volume in patients with first-episode schizophrenia: DARTEL automated analysis and ROI validation. *NeuroImage*.

[57] Ripollés P., Marco-Pallarés J., de Diego-Balaguer R. (2012). Analysis of automated methods for spatial normalization of lesioned brains. *NeuroImage*.

[58] Ashburner J., Friston K. J. (2000). Voxel-based morphometry—the methods. *NeuroImage*.

[59] Gitelman D. R., Ashburner J., Friston K. J., Tyler L. K., Price C. J. (2001). Voxel-based morphometry of herpes simplex encephalitis. *NeuroImage*.

[60] Friston K. J., Holmes A. P., Worsley K. J., Poline J.-P., Frith C. D., Frackowiak R. S. J. (1994). Statistical parametric maps in functional imaging: a general linear approach. *Human Brain Mapping*.

[61] Meinzer M., Beeson P. M., Cappa S. (2013). Neuroimaging in aphasia treatment research: consensus and practical guidelines for data analysis. *NeuroImage*.

[62] Wilke M., Lidzba K. (2007). LI-tool: a new toolbox to assess lateralization in functional MR-data. *Journal of Neuroscience Methods*.

[63] McLaren D. G., Ries M. L., Xu G., Johnson S. C. (2012). A generalized form of context-dependent psychophysiological interactions (gPPI): a comparison to standard approaches. *NeuroImage*.

[64] Friston K. J., Buechel C., Fink G. R., Morris J., Rolls E., Dolan R. J. (1997). Psychophysiological and modulatory interactions in neuroimaging. *NeuroImage*.

[65] O'Reilly J. X., Woolrich M. W., Behrens T. E. J., Smith S. M., Johansen-Berg H. (2012). Tools of the trade: psychophysiological interactions and functional connectivity. *Social Cognitive and Affective Neuroscience*.

[66] Bartels A., Zeki S. (2005). The chronoarchitecture of the cerebral cortex. *Philosophical Transactions of the Royal Society B: Biological Sciences*.

[67] Benjamini Y., Hochberg Y. (1995). Controlling the false discovery rate: a practical and powerful approach to multiple testing. *Journal of the Royal Statistical Society: Series B*.

[68] Genovese C. R., Lazar N. A., Nichols T. (2002). Thresholding of statistical maps in functional neuroimaging using the false discovery rate. *NeuroImage*.

[69] Mechelli A., Price C. J., Friston K. J., Ashburner J. (2005). Voxel-based morphometry of the human brain: methods and applications. *Current Medical Imaging Reviews*.

[70] Allendorfer J. B., Kissela B. M., Holland S. K., Szaflarski J. P. (2012). Different patterns of language activation in post-stroke aphasia are detected by overt and covert versions of the verb generation fMRI task. *Medical Science Monitor*.

[71] Weiduschat N., Thiel A., Rubi-Fessen I. (2011). Effects of repetitive transcranial magnetic stimulation in aphasic stroke: a randomized controlled pilot study. *Stroke*.

[72] Marangolo P., Fiori V., Calpagnano M. A. (2013). tDCS over the left inferior frontal cortex improves speech production in aphasia. *Frontiers in Human Neuroscience*.

[73] Ren C.-L., Zhang G.-F., Xia N. (2014). Effect of low-frequency rTMS on aphasia in stroke patients: a meta-analysis of randomized controlled trials. *PLoS ONE*.

[74] Bates K. A., Rodger J. (2015). Repetitive transcranial magnetic stimulation for stroke rehabilitation-potential therapy or misplaced hope?. *Restorative Neurology and Neuroscience*.

[75] Thiel A., Schumacher B., Wienhard K. (2006). Direct demonstration of transcallosal disinhibition in language networks. *Journal of Cerebral Blood Flow and Metabolism*.

[76] Uruma G., Kakuda W., Abo M. (2010). Changes in regional cerebral blood flow in the right cortex homologous to left language areas are directly affected by left hemispheric damage in aphasic stroke patients: evaluation by Tc-ECD SPECT and novel analytic software. *European Journal of Neurology*.

[77] Rosso C., Perlbarg V., Valabregue R. (2014). Broca's area damage is necessary but not sufficient to induce after-effects of cathodal tDCS on the unaffected hemisphere in post-stroke aphasia. *Brain Stimulation*.

[78] Saur D., Hartwigsen G. (2012). Neurobiology of language recovery after stroke: lessons from neuroimaging studies. *Archives of Physical Medicine and Rehabilitation*.

[79] He B. J., Snyder A. Z., Vincent J. L., Epstein A., Shulman G. L., Corbetta M. (2007). Breakdown of functional connectivity in frontoparietal networks underlies behavioral deficits in spatial neglect. *Neuron*.

[80] Fox M. D., Snyder A. Z., Vincent J. L., Corbetta M., Van Essen D. C., Raichle M. E. (2005). The human brain is intrinsically organized into dynamic, anticorrelated functional networks. *Proceedings of the National Academy of Sciences of the United States of America*.

[81] Fox M. D., Corbetta M., Snyder A. Z., Vincent J. L., Raichle M. E. (2006). Spontaneous neuronal activity distinguishes human dorsal and ventral attention systems. *Proceedings of the National Academy of Sciences of the United States of America*.

[82] Power J. D., Cohen A. L., Nelson S. M. (2011). Functional network organization of the human brain. *Neuron*.

[83] Turken A. U., Dronkers N. F. (2011). The neural architecture of the language comprehension network: converging evidence from lesion and connectivity analyses. *Frontiers in Systems Neuroscience*.

[84] van Hees S., McMahon K., Angwin A., de Zubicaray G., Read S., Copland D. A. (2014). A functional MRI study of the relationship between naming treatment outcomes and resting state functional connectivity in post-stroke aphasia. *Human Brain Mapping*.

[85] Tsai P.-Y., Wang C.-P., Ko J. S., Chung Y.-M., Chang Y.-W., Wang J.-X. (2014). The persistent and broadly modulating effect of inhibitory rTMS in nonfluent aphasic patients: a sham-controlled, double-blind study. *Neurorehabilitation and Neural Repair*.

[86] Seghier M. L. (2008). Laterality index in functional MRI: methodological issues. *Magnetic Resonance Imaging*.

[87] Friston K. (2012). Ten ironic rules for non-statistical reviewers. *NeuroImage*.

