# Supplementary material for: Interhemispheric Plasticity following Intermittent Theta Burst Stimulation in Chronic Poststroke Aphasia
Source: Neural Plast. 2016 Jan 10;2016:4796906. doi: 10.1155/2016/4796906 (PMC4736997; doi:10.1155/2016/4796906)
Supplement: Supplementary file 1 — The Supplementary Material show raw BOLD time-series from each ROI for both patients with lesion-ROI overlaps in Supplementary Figure 1. The purpose of this figure is to illustrate that the time-series from the left IFG ROI, although it overlaps partially with the lesion, shows phasic responses that are similar to those observed for the right IFG ROI (where there is no lesion overlap). In addition, correlational analyses are provided to show that for both patients, the left IFG ROI time-series was more strongly correlated with the right IFG ROI than with the CSF time-series, and that the right-left IFG correlation was only marginally affected by removal of the CSF signal. These analyses demonstrate that the ROI-lesion overlap was unlikely to introduce artifactual effects into the left IFG signal. [file 4796906.f1.docx]

Supplementary S1

**A.**

**B.**

Demeaned IFG ROI timeseries for patients P1(A) and P4(B) from pre-iTBS scans are shown to illustrate pre-iTBS activation patterns in each hemispheric ROI.

Both patients showed weak correlations between CSF timeseries and left IFG timeseries at baseline (P1 r=0.23; P4 r=0.13) but not between CSF timeseries and R IFG timeseries (P1 r =0.08; P4 r=-0.01) at baseline. Both patients showed moderate correlations between left IFG timeseries and right IFG timeseries (P1 r = 0.41; P4 r= 0.37) that were only marginally affected by the removal of CSF timeseries (P1 partial r = 0.40; P4 partial r = 0.38) at baseline. At follow-up, P1 showed weak correlations between CSF timeseries and the left IFG ROI timeseries (P1 left IFG r = 0.16, right IFG r=0.04), and P4 showed weak correlation between CSF timeseries and both left and right IFG ROI timeseries (P4 left IFG r = 0.18, right IFG r = 0.14). Moderate timeseries correlations between left and right IFG ROIs were also observed at follow-up (P1 r = 0.29; P4 r= 0.39) that were only marginally affected by the removal of CSF timeseries (P1 partial r = 0.28; P4 partial r = 0.39). While gPPI models for all patients included CSF covariates for quality control, the contribution of CSF signals did not substantially influence the overall relationship between left IFG/right IFG timeseries at either scan session for the two patients with lesion-ROI overlaps.
